# Chemical and Immunological Characteristics of Aluminum-Based, Oil-Water Emulsion, and Bacterial-Origin Adjuvants

**DOI:** 10.1155/2019/3974127

**Published:** 2019-05-08

**Authors:** Susana Martiñón, Angel Cisneros, Sergio Villicaña, Ricardo Hernández-Miramontes, Edgar Mixcoha, Psyché Calderón-Vargas

**Affiliations:** ^1^Neuropharmacology, Biotechnology and Experimental Therapeutic Anti-Addictions Laboratory, National Institute of Psychiatry Ramón de la Fuente Muñiz, Mexico City, Mexico; ^2^Faculty of Health Sciences, Anahuac University, Huixquilucan, Estado de Mexico, Mexico; ^3^CONACYT Researcher Fellowship-National Institute of Psychiatry Ramón de la Fuente Muñiz, Mexico; ^4^Centro de la Conducta S.C., Tijuana, Baja California, Mexico

## Abstract

Adjuvants are a diverse family of substances whose main objective is to increase the strength, quality, and duration of the immune response caused by vaccines. The most commonly used adjuvants are aluminum-based, oil-water emulsion, and bacterial-origin adjuvants. In this paper, we will discuss how the election of adjuvants is important for the adjuvant-mediated induction of immunity for different types of vaccines. Aluminum-based adjuvants are the most commonly used, the safest, and have the best efficacy, due to the triggering of a strong humoral response, albeit generating a weak induction of cell-mediated immune response. Freund's adjuvant is the most widely used oil-water emulsion adjuvant in animal trials; it stimulates inflammation and causes aggregation and precipitation of soluble protein antigens that facilitate the uptake by antigen-presenting cells (APCs). Adjuvants of bacterial origin, such as flagellin, *E. coli* membranes, and monophosphoryl lipid A (MLA), are known to potentiate immune responses, but their safety and risks are the main concern of their clinical use. This minireview summarizes the mechanisms that classic and novel adjuvants produce to stimulate immune responses.

## 1. Introduction

Vaccines constitute one of the greatest achievements in the history of medicine, their main objective being the prevention of diseases by inducing the immune response. Purified antigen-based vaccines, either synthetic or recombinant, are more specific but less immunogenic than original vaccines formed by live attenuated or inactivated microbes; therefore, associated agents called “adjuvants” are required, which increase their immune response strength, quality, and duration (memory) [1].

Adjuvants aim, ideally, to increase and improve the immunogenicity of antigens by decreasing the amount and number of immunizations; thus, the search for new substances with adjuvant/immunopotentiating activity has been one of the main trends in immunological research for over a decade [2]. The rational design of vaccines involves the logical choice of the immunopotentiator, based on their mode of action and its expected effect on the efficacy and safety of the vaccine [3].

In this paper, we address the use of the most frequently used adjuvants in experimental and clinical trials, describing their immunological characteristics, with the objective of helping the decision on the best adjuvant for each vaccine.

## 2. Adjuvants

One of the most important issues when designing efficient and safe vaccines is the selection of an appropriate adjuvant that meets both the desired immunogenic potential and the safety requirements for human use. The best adjuvant is considered to be the one that elicits the most potent immune response while posing the least risk to the individual's health.

Adjuvants, or immunopotentiating agents, are a group of substances with divergent chemical structures that are used to increase, improve, or extend the immune response against a simultaneously administered antigen [2, 4]. The concept was pioneered in the 1920s by Ramon [5–7] who pointed out that horses that developed an abscess at the diphteria toxoid inoculation site produced higher specific antibody titers than those who did not.

Adjuvants are normally used with several purposes: (a) to improve the immunogenicity of highly purified or recombinant antigens; (b) to reduce the amount of antigen or the number of vaccine administrations required for the development of immunity; (c) to improve the efficiency of vaccines in newborns, the aged, and immunocompromised individuals; and (d) to be used as systems for the delivery of the antigen and its assimilation in mucosa [5].

**Figure 1:**
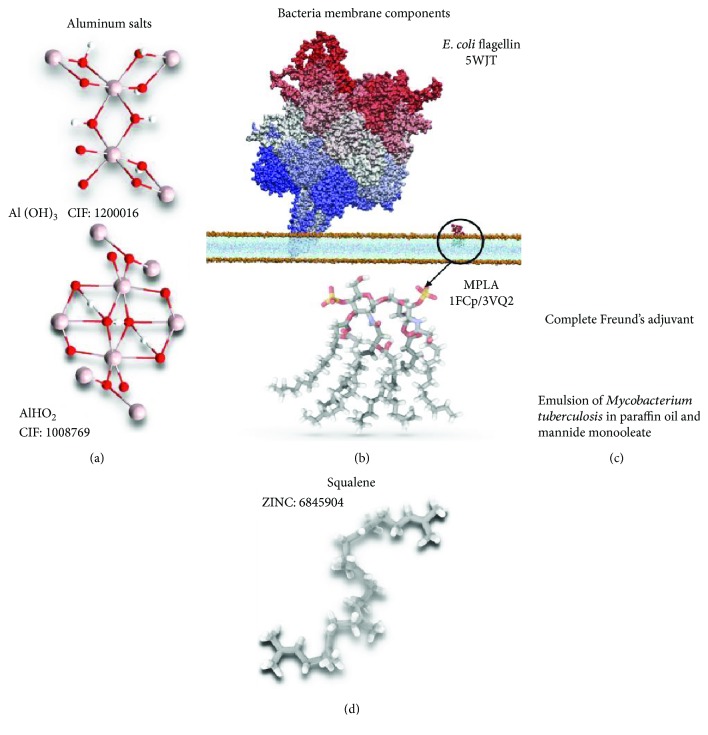
Three-dimensional representation of adjuvants. (a) Crystal structures of aluminum salts used as adjuvants in human vaccines. Al(OH)_3_ is the most widely used adjuvant in some crystal structures (such as gibbsite) and amorphous forms [86]. Another aluminum salt used in vaccines is aluminum oxide hydroxide such as goethite [87]. (b) Several bacterial membrane proteins are used as adjuvants in order to activate human immune cells. Bacterial flagellin is detected by TLR5 in innate cells activating a high immune response; recently, the *B. subtilis* flagellin structure was solved using cryomicroscopy under the 5WJT PDB code [88]. On the other hand, phospholipids and lipidic components in the bacterial membrane are recognized as dangerous and activate immune response. (c) The most used adjuvant in animal immunization is an emulsion of oil, paraffin, and *M. tuberculosis* death cells. (d) Squalene is an oil compound present in the liver of sharks as a precursor of cholesterol metabolism. In recent years, squalene has been accepted as an adjuvant for human vaccination. Immunological results of squalene have demonstrated it to be an efficient adjuvant. The coordinates of squalene were taken from the ZINC15 data bank [89].

The most used adjuvants in clinical and experimental trials are enunciated in Figure 1, and the chemical and immunological characteristics of each one are described as follows.

## 3. Aluminum-Based Adjuvants

Aluminum-based adjuvants are used in at least 146 approved vaccines for the prevention of disease, which make them the most commonly used [8]. As a matter of fact, until 1997, aluminum-based adjuvants were the only ones approved for use in humans and remain to be the most stable, safe, tolerated, and effective. The most used aluminum-based adjuvants in vaccines are salts of three types: aluminum hydroxide, aluminum phosphate, and potassium aluminum sulfate [9].

Aluminum hydroxide gels are slightly crystalline and amorphous and have a mineral structure of pseudoboehmite, oxy-based aluminum hydroxide, and AlO(OH)·nH_2_O. Gels comprise both micro- and nanoparticles, formed by aggregates of primary crystals of up to 10 nm in length [10]. Oxyaluminum hydroxide has a surface area of 500 m^2^/g, and only the outer layer is antigen-associated by surface adsorption; the association is carried out mainly by electrostatic forces, hydrophobic interactions, hydrogen bonds, ligand exchange, and Van der Waals forces. The point of zero charge (PZC) is 11.4, so in a neutral pH, it is positively charged, allowing antigens to bind primarily to their negative charges. Additionally, either PZC or antigen binding can be altered in both power and stability, combining it with phosphate counterions [8–10].

The mechanisms of action of aluminum hydroxide and, in general, aluminum-based adjuvants include (1) aggregate formation enabling continuous release of antigens; (2) formation of particle structures that promote phagocytosis of antigens by antigen-presenting cells (APC); and (3) induction of local inflammation via the NLRP3 inflammasome, which results in the recruitment and activation of macrophages and increase in the expression of molecules of major histocompatibility complex (MHC) class II and antigen presentation [11]. The activation of the inflammasome induces the secretion of mature IL-1*β* and IL-18 by dendritic cells and the differentiation of TH2 cells, promoting the activation of B cells and the subsequent production of antibodies, predominantly IgG [12, 13] [14]. However, NLRP3-independent antibody production pathways have been shown, as well as a nonphagocytic way of acting of the aluminum hydroxide [13].

Summarizing, aluminum-based adjuvants trigger a strong humoral immune response primarily mediated by secreting antibodies specific to antigens, particularly IgG1, albeit generating a weak induction of cell-mediated immune response [14].

## 4. Oil-Water Emulsion Adjuvants

### 4.1. Freund's Adjuvant

The most widely used oil-water emulsion adjuvant in animal experimentation is Freund's adjuvant, from which there are two variants: the incomplete (Incomplete Freund's Adjuvant (IFA)) and the complete (Complete Freund's Adjuvant (CFA)) adjuvant [15]. For enhancing the immune response, the CFA contain killed mycobacteria (*Mycobacterium tuberculosis*) which are responsible for attracting macrophages and other cells to the site of injection, and due to that, it is usually applied in the initial immunizations. Due to its toxicity and secondary reactions, the use of CFA in humans is ineligible as a proper adjuvant; however, IFA is less toxic and therefore suitable for its clinical usage [16].

The CFA is used to prepare oil-water emulsion adjuvants with the immunogen so that the antigen is slowly released and produces a high and long duration stimulation of the immune response. A typical composition of CFA comprises 1 mg *Mycobacterium tuberculosis* heat killed and dried with 0.85 mL paraffin and 0.15 mL of Arlacel® 83 (a mixture of oleic, palmitic, stearic, and linoleic esters with 2-(3,4 dihydroxytetrahydrofuranyl)-ethylene glycol) [17]. The *M. tuberculosis* immune effects will be reviewed in Adjuvants of Bacterial Origin.

Common pathways of antigen and adjuvant emulsion inoculation are intradermal, subcutaneous, and intramuscular, although the intraperitoneal pathway is also used [18]. It has the great disadvantage such that its use is restricted only for laboratory animals because it contains mineral oil that is not metabolized by humans and the mycobacterial elements can lead to granulomatous reactions [4].

### 4.2. Squalene

Squalene is a terpene found in plants and the liver of some animal species, including humans. It acts as a precursor for cholesterol, steroid hormones, and vitamin D. Squalene for commercial purposes is usually extracted from shark liver oil, but it can also be obtained from vegetable oils, such as olive oil and palm oil [19, 20]. There were reports by Asa et al. [21] that squalene-based vaccines could lead to the production of anti-squalene antibodies [22, 23], although these claims were later criticized and have been a controversial subject [22, 24, 25]. Nevertheless, more than 20 million doses of squalene-based vaccines have been administered worldwide with biosafety results ranging from acceptable to excellent [25, 26].

Several adjuvants containing squalene have been used in licensed human vaccines, such as MF59, which is an oil-in-water nanoemulsion containing squalene, and Tween 80 and Span 85 (both surfactants); AS03 and AS04, containing *α*-tocopherol (a form of vitamin E); and polysorbate 80 [27]. Squalene is used as an adjuvant for several different vaccines along with antigens from the influenza virus, hepatitis B and C viruses, the herpes simplex virus, etc. [25]. MF59 has been associated with an increase in the activity of helper T lymphocytes and higher concentrations of immunoglobulins, specifically the IgG1 and IgG2a isotypes. MF59 has also been reported as capable of inducing Th1 responses in CD8^+^ T lymphocytes [26].

Recent studies have shown that the MF59 adjuvant contributed to seroconversion and seroprotection 21 days after the first administration of an influenza A H1N1 vaccine. The same vaccine elicited higher percentages of seroprotection and seroconversion after 21 and 42 days when adjuvanted than when nonadjuvanted. Regarding the safety of the vaccine, this cohort reported mild to moderate adverse effects, including pain and bruising at the injection site, as well as muscular ache. Nevertheless, none of these adverse reactions lasted beyond 72 hours [28].

### 4.3. Other Squalene-Based Adjuvants

Formulations including squalene and other compounds have also been tested. GLA-SE is an oil-in-water emulsion with squalene and glucopyranosyl lipid adjuvant (GLA). This formulation has been shown to induce strong signaling through the TLR-4, caspase, IL-18, and IFN-*γ* pathways, leading to a Th1 response [29]. GLA-SE has been used as the adjuvant for a tuberculosis vaccine in humans with potent antibody responses peaking after the second immunization and mild side effects including headaches and fatigue [30]. MPL-SE is a mix of MPL-A (a nontoxic derivative of the lipopolysaccharide of *Salmonella minnesota*; see Monophosphoryl Lipid A (MPL-A)) with squalene oil, excipients, and water. As it represents an excellent promoter for Th1 responses, there is ongoing research regarding its applicability for leishmaniasis vaccines [26]. Syntex Adjuvant Formulation (SAF) is an oil-in-water emulsion that contains squalene, Tween TM 80, and Pluronic TM L121 in phosphate-buffered saline. It is currently in preclinical tests for vaccines containing antigens from the influenza virus, the Epstein-Barr virus, and the Human Immunodeficiency Virus (HIV) [26].

## 5. Adjuvants of Bacterial Origin

### 5.1. Flagellin

Flagellin is a protein composed of 494 amino acids and is the most important structural protein in the flagella of Gram-negative bacteria [31]. It is composed of three domains (D1, D2, and D3), with D1 and D2 being highly conserved and D3 being hypervariable [32]. As the primary component of flagella, it contributes to motility of bacterial cells. Most of the bacterial flagellin molecules stay in their flagella, but some of them are released to the medium, enabling its recognition by the immune system.

It has been reported that the hyperconserved N and C termini of flagellin can be recognized by Toll-like receptor (TLR) molecules, particularly by TLR-5 [33–35]. This ability of flagellin to stimulate TLR-5 makes it an interesting option as an adjuvant. Flagellin concentrations in the range of 1 to 10 nM elicit the maximal intensity of TLR-5 signaling. TLR-5 is expressed on different kinds of cells, which include monocytes, macrophages, neutrophils, lymphocytes, NK cells, and dendritic cells. Recognition of TLR-5 with flagellin leads to signaling via both MyD88-dependent and MyD88-independent pathways. These pathways lead to the induction of transcription factors AP-1, NF-*κ*B, and IRF3 [32]. These, in turn, trigger the production of cytokines and chemokines that recruit dendritic cells, T lymphocytes, and B lymphocytes to lymph nodes [31]. Flagellin can also promote strong Ag-specific CD4^+^ T-cell responses by interacting with TLR-5 on CD11c^+^ cells. As this results in high antibody titers, flagellin exhibits potential as an adjuvant [36, 37].

Flagellin has been used as an adjuvant by joint administration with the main antigen and through fusion proteins resulting from the addition of epitopes linked to the flagellin molecule. Several studies have been made in order to determine in which regions of the flagellin molecule the epitopes should be inserted to maximize antibody titers, but no definitive conclusions have been reached. Song et al. obtained optimal antibody titers by introducing a hemagglutinin epitope in the hypervariable region of flagellin [38]. Other studies reported that inserting L1R epitopes in the hypervariable region does not produce antibodies [31] and that inserting L1R epitopes in the N-terminus of flagellin could lead to antibodies that interact with the native L1R [39]. Alternatively, Lin et al. showed that insertion of epitopes towards the C-terminus of flagellin can induce signaling via NLRC4 and NAIP5, leading to CD8^+^ T-cell responses against tumor cells [40]. This variety of responses exhibits the versatility of flagellin as an adjuvant for different purposes depending on the region of antigen insertion, but further studies are required to fully harness this potential.

The use of flagellin as an adjuvant has several advantages regarding safety: only low doses are required for it to be effective, it does not elicit the synthesis of IgE, no toxicity has been associated with its intranasal administration in animal models, and it can be easily produced in large quantities. Nevertheless, studies in humans are still in phase I and have not been conclusive regarding the adverse effects that could be derived from its use [31]. A study involving flagellin fusion proteins for an influenza A H1N1 vaccine reported that some individuals showed systemic adverse effects. However, some of them were stabilized after 4 days of rest, and another one did so after taking nonsteroidal anti-inflammatory drugs (NSAIDs) [41].

### 5.2. Bacterial Membranes

*E. coli* is the most widely studied Gram-negative prokaryotic microorganism in numerous areas of science. The structure of its membrane, as shown in Figure 1, has several relevant features that can be used in the field of immunology and the design of vaccines and adjuvants. As it is a Gram-negative bacterium, its cell wall has an inner layer that is composed of peptidoglycan that comprises only 10% of the whole structure, whereas most of the cell wall is formed by an outer membrane. The outer membrane is composed of phospholipids and proteins, just like the cytoplasmic membrane, as well as polysaccharides. Lipids and polysaccharides in the outer membrane are usually bound and form a complex called lipopolysaccharide (LPS), which is toxic for animals. The polysaccharides in LPS and polysaccharide O comprise its core, whereas its lipid component is known as lipid A [42]. A noteworthy feature of Gram-negative bacteria is the release of outer membrane vesicles (OMVs). OMVs are spherical, nanometric vesicles that are released during normal growth and are formed by protuberances in the outer membrane, so they contain LPS, peptidoglycans, phospholipids, and proteins [43, 44].

OMVs represent a novel approach for the development of vaccine adjuvants because of their inherent inflammatory potential, as they stimulate the immune innate system. They activate simultaneously the humoral response and both CD4^+^ T-cell and B-cell responses [43–45]. This mechanism is driven primarily by recognition of LPS and other molecules on membranes by TLR molecules and the complement system [44, 46, 47], leading to the recruitment of antigen-presenting cells [48]. The joint stimulation by the molecules present in OMVs results in a more potent response than that elicited by LPS on its own [49, 50], which makes OMVs attractive as adjuvants. Another immunogenic molecule present in bacterial membranes is protein D, which has been used as an adjuvant for a vaccine against *Haemophilus influenzae* [51].

Different approaches have been proposed to use OMVs as adjuvants. These include joint administration [52], adding desired epitopes to proteins displayed on the surface of the OMVs [53, 54], and delivering proteins within the OMVs [55]. Antibody titers have been higher for the fusion proteins displayed on the surface of the OMVs [43], suggesting vaccines that expose the target epitope could be more successful.

Several vaccines have been put forward using OMVs as adjuvants. Early examples include vaccines against *Neisseria meningitidis*, which have shown an effectiveness of 73% or higher in different cohorts [56–58]. These vaccines have been used to fight epidemics and have been part of vaccination programs for more than 20 years [59, 60]. There are some other vaccines that use OMVs and have started moving to clinical trials because of their safety and effectiveness. These include vaccines against allergens (phase I clinical trials) [61, 62], *Shigella flexneri* (phase I and II clinical trials) [63, 64], and influenza [65]. These vaccines have been administered intranasally and have elicited antibody production with very minor side effects, which highlights the potential of OMVs as adjuvants. Moreover, immunostimulatory proteins and LPS from OMVs do not replicate, which increase their safety [43, 44].

Nevertheless, the use of OMVs as adjuvants poses several challenges regarding production and design. Their mass production would be a complex procedure since their content of some endotoxins must be monitored to avoid excess inflammation [66–68]. In particular, the dose of LPS must be controlled accurately because OMVs with low LPS are less-effective adjuvants, while excessive LPS can cause toxic effects [69, 70].

### 5.3. Monophosphoryl Lipid A (MPL-A)

LPS is composed of three different regions, namely, lipid A, the core, and a specific glycan. Lipid A is noteworthy because it is responsible for anchoring LPS to the outer membrane and for the endotoxic activity of LPS [71]. Toxicity of lipid A is elicited by the potent stimulation of TLR-4 and intracellular signaling that activates caspases [72, 73]. The toxic capability of lipid A can be diminished by means of some structural changes, such as removal of the C1-glucosamine phosphate group, which yields monophosphoryl lipid A (MPL-A) [8]. MPL-A has been shown to induce maturation of dendritic cells, CD4^+^ T-cell clonal expansion, and Th1 responses without the inflammatory effects of LPS [68, 74]. However, CD4^+^ T-cell clonal expansion and Th1 differentiation induced by MPL-A is not as long-lasting as that induced by LPS, as T-cell counts induced by MPL-A are lower than those induced by LPS after 21 days [68]. Other studies have shown that MPL-A induces JNK- and mTOR-dependent signaling in macrophages and dendritic cells. This pathway leads to increases in the metabolic activity of macrophages for antimicrobial purposes [75] and the production of proinflammatory cytokines in dendritic cells [76].

MPL-A obtained from *Salmonella minnesota* was historically the first TLR ligand to be approved for use in humans as an adjuvant [26]. However, MPL-A by itself is not water soluble, which has led to the development of different vehicles to increase its bioavailability after administration. Some strategies that have been explored are the adsorption of MPL-A by aluminum hydroxide molecules and the delivery of MPL-A in liposomes [8]. Both formulations offer advantages. The formulation of MPL-A adsorbed by aluminum hydroxide (ASO4) elicits a higher antibody response with fewer doses than aluminum hydroxide by itself [77]. In turn, liposomes have been successful as adjuvants of vaccines that use immunogenic carrier proteins to induce an immune response against a hapten, that is, a molecule that normally would not induce an immune response [78] and DNA vaccines [79].

Clinical trials for vaccines with MPL-A have been successful. A vaccine for human papilloma virus (HPV) elicited high antibody titers and had only injection site reactions as adverse effects. Antibody titers were particularly high for adolescent girls, suggesting there is an ideal age for administration of the vaccine [77]. Another study showed that the use of liposomes containing MPL-A and adsorbed by aluminum hydroxide could elicit immune responses against repeat-based malaria antigens [80]. However, a potential drawback of the large-scale production of MPL-A for vaccines is that it is obtained through extensive processing of LPS. This leads to large variability between batches and could compromise the efficiency of vaccines [81].

#### 5.3.1. Mycobacterium Tuberculosis

In Oil-Water Emulsion Adjuvants, the CFA was mentioned; however, since it is complemented with *Mycobacterium tuberculosis*, its principal mechanism of action must be mentioned in this section.

The active components conferred by mycobacteria are a dipeptide, N-acetylmuramyl-L-alanine-D-isoglutamine (MDP), a molecule that activates macrophages and dendritic cells through the nucleotide-binding oligomerization domain containing 2 (NOD2) and skeletal elements of the bacterial cell wall [82, 83]. Besides stimulating inflammation, adjuvants cause aggregation and precipitation of soluble protein antigens to form particles that facilitate their efficient uptake by APCs. The particulate nature of the antigen also reduces the speed with which the antigen is removed from the system, and this action favors the inflammasome activation [84]. The CFA promotes Th1 subpopulation, promotes synthesis of IgG rather than IgM, inhibits the induction of tolerance, and favors delayed hypersensitivity reactions [85].

## 6. Discussion

The selection of the “best adjuvant” is relative to the goal of the use; i.e., it will be the one that helps to develop an immune response according to the needs of the antigen of interest. As discussed above, some objectives require the robust production of antibodies [25, 28, 38, 61, 63–65, 78]. In such cases, adjuvants such as squalene or even flagellin help the fusion of proteins with the epitope of interest in the hypervariable region OMV, and liposomes carrying MPL-A have been primarily explored because of the immune responses they elicit. Alternatively, other cases [26, 40] require a cytotoxic T-cell response, which is better elicited by epitopes of interest inserted at the C-terminus of flagellin or adjuvants like squalene oil-in-water emulsions. Thus, adjuvant selection is a critical step in vaccine or immunotherapy design.

Equally important, the adjuvant needs to be safe and should have the least intense adverse reactions or, preferably, that it does not have them. Successful adjuvants for vaccines should be easy to access with low cost, as to guarantee that they can be used in any final population that requires the vaccine to be developed. Finally, it is desirable that the adjuvant is applied only once. This review offers chemical and immunological characteristics of the most popular adjuvants. However, it is a very broad area of research, which requires more studies and the invention of new pharmacological formulations, either combinations of adjuvants already in use or of new molecules.

## 7. Conclusion

Adjuvants are powerful elements that help with the development of robust immune responses to vaccines. The selection of an adjuvant for each type of vaccine must be made by clearly defining its objective. This simple choice can and will favor the best choice to improve the functionality of future vaccines against numerous diseases.
